# Assessment of Non-physical User Violence and Burnout in Primary Health Care Professionals. The Modulating Role of Job Satisfaction

**DOI:** 10.3389/fpubh.2022.777412

**Published:** 2022-02-04

**Authors:** David Pina, Paloma Llor-Zaragoza, Reyes López-López, Jose Antonio Ruiz-Hernández, Esteban Puente-López, Inmaculada Galián-Munoz, Begoña Martínez-Jarreta

**Affiliations:** ^1^University of Murcia, Department of Socio-Sanitary Sciences, Murcia, Spain; ^2^Applied Psychology Service, University of Murcia, Murcia, Spain; ^3^Department of Psychiatry and Social Psychology, University of Murcia, Murcia, Spain; ^4^Administration of the National Institute of Social Security (INSS), Ministry of Work, Migration and Social Security, Murcia, Spain; ^5^Department of Pathological Anatomy, Forensic and Legal Medicine and Toxicology, University of Zaragoza, Zaragoza, Spain

**Keywords:** primary health care (PHC), violence, burnout - professional, psychology, job satisfaction

## Abstract

**Introduction:**

Growing concern about workplace violence shows the need for an evaluation in specific contexts in order to identify the particularities of each professional group. The health sector consists of a group of professionals with high exposure to violence, specially from users. There are differences depending on the professional category or unit in which the professional works. In this regard, Primary Health Care (PHC) is characterized by a personalized and continuous patient treatment over time, which is not exempt from cases of violence. Among the commonly studied consequences of these situations are decreases in job satisfaction and burnout.

**Objective:**

The main objective of this study is to analyze the modulating role of job satisfaction in the relationship between non-physical user violence and the onset of burnout.

**Methods:**

Cross-sectional comparative descriptive design. The sample consisted of 574 professionals from 39 PHC centers of the Murcian Health Service. Data were collected using two-stage cluster sampling. For data analysis, descriptive analysis, correlations and stepwise hierarchical regression were used to analyze the interaction between the variables.

**Results:**

Regression analysis draws a model where non-physical violence and low intrinsic and extrinsic job satisfaction act as modulators of non-physical violence, cynicism and emotional exhaustion.

**Conclusions:**

This study provides evidence of the psychological consequences of the perception of user violence in the PHC staff. Furthermore, it is evident that the emergence of burnout syndrome in these professionals is related to exposure to verbal or non-physical violence together with low job satisfaction. In this sense, a circular and bidirectional relationship between the variables studied is proposed as a possible explanatory model.

## Introduction

Violence is present in virtually all areas of human activity, having multiple social, legal and public health implications (1, 2).

One context where violence has been extensively studied is in the workplace, where it has been defined as: “*incidents in which a worker suffers mistreatment, threats or attacks in circumstances related to their work and which, implicitly or explicitly, endanger their safety, well-being, or health*” (3). If we look at the way in which it manifests, we could classify workplace violence as non-physical violence (verbal abuse, threats, ironic language, derogatory glances, provocative or aggressive body language) and physical violence (physical intimidation and damage to persons, property or furniture) (2, 4).

Unlike most professions, in which workplace violence is more frequent among co-workers, in the health sector, it has been observed that violence received from users is even greater than the violence received from co-workers, although both are present (5, 6). Within this work group, even though there is no consensus in the literature, there are differences between medical and nursing professionals (6–8). Differences between health care and support staff have also been observed, the latter being more exposed (8).

Among the manifestations of violence, physical violence is much less common. Only 11% of health care workers report having suffered at least one episode of physical aggression, whereas non-physical aggressions affect approximately 6 out of 10 professionals (9, 10). Due to its high prevalence, the present study will focus on non-physical violent behavior.

The service to which the professional belongs also seems to be a relevant variable. A higher number of violent incidents with users have been observed in the Emergency and Psychiatry Units (11, 12) where physical aggressions are between 48 and 26.9% and non-physical between 87.2 and 58.6% (11). Despite not being one of the sectors with the highest incidence, our study focuses on Primary Health Care (PHC) professionals, which, although it is a service characterized by close and continuous treatment of the patient, it has been observed that they may also be exposed to a high frequency of violent behavior, predominantly non-physical violence (8, 13, 14).

What has been described so far evidences that health care professionals are highly exposed to violence in the workplace. This exposure may have a negative impact on their health, job satisfaction or emotional exhaustion and stress at work. Recently, it has been observed that at least five out of ten professionals show a high degree of emotional exhaustion, six out of ten show a high degree of depersonalization and six out of ten show a low degree of self-fulfillment (15). Other studies found that 26.7% of participants had high emotional exhaustion, 38.1% had high cynicism and 35.6% had low self-fulfillment (16). Specifically, in Spain, where the present study was carried out, 23% emotional exhaustion, 11.4% cynicism and 12.8% reduced self-fulfillment were observed in health professionals (17).

These symptoms, congruent with a state of burnout, have also been associated with socio-demographic factors of the professionals (15, 18). In this regard, being young (19) or having more professional experience seem to be influential factors. From the perspective of psychological well-being, having symptoms of depression, managing aggression through anger, having a lower perceived self-efficacy, having the feeling of not progressing professionally or a bad working environment were significantly associated with emotional exhaustion and cynicism (15).

Another particularly relevant aspect is job satisfaction, both from a perspective of intrinsic factors (responsibility, achievements, recognition, etc.) and extrinsic factors (salary, physical environment, safety, etc.). In this sense, the systematic review conducted by Lu et al. (20) points out as satisfaction sources working conditions, interactions (relationships with patients, co-workers and managers), the work itself (workload, scheduling, challenging work, routinization, task requirements), remuneration (pay, salary), self-development and promotion (professional training, promotion opportunities, job promotion, personal achievements), praise and recognition, control and responsibility, autonomy, decision-making, job security, leadership styles and organizational policies. The prevalence of job dissatisfaction among health professionals varies depending on the place of origin of the study. For example, the study by Aiken et al. (21) found the highest nurses' job dissatisfaction in the United States (41%), although later studies found a higher percentage in Greece (56%) (22), Ethiopia (46.8%) (23) and Ireland (42%) (22). In Spain, the study by Aiken et al. (22) reports a 38% dissatisfaction rate among Spanish nursing professionals. These numbers are of importance because several studies have identified this variable as a key factor when dealing with situations with a violent user, as well as for improving performance and patient care, reducing sick leave, increasing motivation, autonomy, leadership, or job promotion, among others (20, 24, 25). With specific regard to violence, a significant relationship has been found between exposure to workplace violence and lower job satisfaction (26–29). In turn, disadvantageous coping with aggression is associated with greater job dissatisfaction (29). Thus, emotional regulation in such situations seems to play an important role in job satisfaction (30).

This context where exposure to violence may be combined with the presence of burnout, low job satisfaction could lead to poorer patient care as well as worse quality indicators or the perception of job insecurity (25, 31–33). Based on this, the present study aims to explore the modulating role of job satisfaction in the relationship between non-physical violence by users and burnout.

## Methods

This study uses an associative cross-sectional design. The starting hypothesis are that high job satisfaction scores will be inversely related to the occurrence of job burnout and non-physical user violence. To this end, we will specifically (1) analyze the perception of non-physical violence and burnout by health professionals and (2) assess the modulating role of job satisfaction.

### Participants

The sample consists of 574 PHC professionals belonging to 39 PHC centers of the Murcian Health Service (southeast of Spain). The majority were women (68.1%), married or living with a partner (72.6%), and with a mean age of 49.6 years (*SD* = 8.4) (see Table 1). Regarding professional category, the sample consisted of medical staff (38.9%), nurses (34%) and non-sanitary staff (25.8%). Finally, 82.6% of the sample had a fixed-indefinite contract and 51.1% of the participants have 10 and less years of job tenure.

**Table 1:** Sociodemographic and occupational characteristics of the sample.

**Variable**	***n***	**%**
Age (years)		
Younger than 35	32	5.6
36–45	143	24.9
46–55	216	37.6
56–65	162	28.2
Missing data	21	3.7
Sex		
Men	178	31
Women	391	68.1
Missing data	5	0.9
Marital status		
Single	90	15.7
Common law partner or married	417	72.6
Divorced, separated, or	58	10.1
widowed	9	1.6
Missing data		
Type of contract		
Permanent	474	82.6
Temporary-Substitution	81	14.1
Missing data	19	3.3
Professional group		
Medical staff	223	38.9
Nursing staff	195	34
Support personnel	148	25.8
Missing data	8	1.3
Job tenure (years)		
0–2	80	14
3–5	102	17.8
6–10	111	19.3
11–15	50	8.7
+15	92	16
Missing data	139	24.2
Professional tenure (years)		
0–10	41	7.1
11–20	108	18.8
21–30	166	28.9
+30	104	18.2
Missing data	155	27

### Instruments

A set of questionnaires was administered summing a total of 58 items, including the sociodemographic and socio-occupational variables described above (age, sex, marital status, type of contract, professional group, tenure in the current position, and tenure in the profession), together with the following variables:

*Healthcare-Workers' Aggressive Behavior Scale-Users-Primary Healthcare* (*HABS-U-PHC*) (34). This instrument assesses the perception of low and medium intensity user violence by professionals in the area of PHC. It consists of 14 items with 5 response options, ranging from 1 (never) to 6 (daily). The items are distributed in two factors: non-physical violence (α = 0.92, 40.61% of explained variance), and physical violence (α = 0.68, 10.59% of explained variance). In the present study, the internal consistency was α = 0.87 for non-physical violence and α = 0.68 for physical violence.

*Overall Job Satisfaction* (*OJS*) (35), adapted to Spanish by Pérez and Hidalgo (36). This scale assesses job satisfaction using 15 items with a response format ranging from 0 (*very dissatisfied*) to 6 (*very satisfied*), grouped into two subscales: Intrinsic satisfaction, which addresses aspects such as acknowledgment of work, responsibility, or promotion (α = 0.85); Extrinsic satisfaction, which explores the organization of the work, the schedule, remuneration, or physical conditions (α = 0.72). In this study, the internal consistency obtained was α = 0.84 for Internal satisfaction and α = 0.69 for External satisfaction.

*Maslach Burnout Inventory-GS* (*MSI-GS*) (37), in the Spanish version of Salanova et al. (38). This scale evaluates burnout through three dimensions: Emotional exhaustion, referring to the loss of emotional resources derived from work (α = 0.90); Cynicism, reflecting indifference and distant attitudes toward work (α = 0.81); and Professional realization, perceived when the work is carried out (α = 0.73). It consists of 16 items with 6 response options ranging from 0 (never) to 6 (daily). In this study, an internal consistency of α = 0.74, was obtained for the subscale of Emotional Exhaustion, α = 0.76 for Cynicism, and α = 0.73 for Professional Realization.

### Procedure

At the time of sample collection, approximately 2,575 professionals were working in 74 PHC centers throughout the Region of Murcia. In order to achieve a 95% confidence level and an assumed error of 3%, a sample size of 510 professionals was estimated to be necessary. A two-stage cluster sampling was used. In the first stage, the population was stratified by PHC centers (clusters), and 39 centers were selected by simple random sampling. Subsequently, for the selection of professionals, a fixed proportion was used, numbering the alphabetical list of all professionals in each center with multiples of three, and finally inviting those selected to participate in the study. In order to reach the estimated number, 670 protocols were distributed, assuming a possible non-response rate of 30%. Meetings were held with the coordinators of the selected centers, in which they were informed of the study, and the research protocol was distributed in printed form. Scheduled visits to the center were made to clarify possible doubts and to compile the completed protocols. The final response rate for this study was 80.60%. Participation was voluntary, ensuring the strictest confidentiality and anonymity of the data collected. All participants were provided with an information sheet and informed consent. The protocol included an informative document together with the approval of the Ethics Committee of the Murcian Health Service.

### Data Analysis

The descriptive statistics of interest, correlations and internal consistency (Cronbach's alpha) of the variables were calculated. To explore the relationships and determine the interaction effect between manifestations of violence and the rest of the study variables, a stepwise hierarchical regression analysis was applied, introducing the independent variables in two successive steps. The first step included one of the dimensions of user violence (non-physical violence) and one of the dimensions of job satisfaction (extrinsic-intrinsic satisfaction). In the second step, the interaction of the independent variables used was introduced. The statistical package SPSS ® version 22 was used for these operations.

For the graphical representation of the results related to the regression analysis, the quartiles were taken as a reference, so that a high value in the variable corresponds to the scores found in Q4, and a low value corresponds to Q1, discarding the Q2 and Q3 values.

## Results

Table 2 shows the descriptive analyses and internal consistencies of the target variables. The dimensions of satisfaction were negatively correlated with violence, emotional exhaustion and cynicism, but positively correlated with professional fulfillment. In addition, non-physical violence was significantly and positively correlated with emotional exhaustion and cynicism.

**Table 2:** Descriptive statistics, reliabilities, and correlations between variables.

**Variables**	***M***	***SD***	**ITEM**	**1**	**2**	**3**	**4**	**5**	**6**	**7**
1. Physical violence	1.13	0.44	3	**0.68**						
2. Non-physical violence	2.29	1.15	11	0.25^**^	**0.87**					
3. Intrinsic satisfaction	3.49	1.16	7	−0.13^**^	−0.35^**^	**0.84**				
4. Extrinsic satisfaction	3.59	0.91	8	−0.08	−0.28^**^	0.77^**^	**0.69**			
5. Emotional exhaustion	2.91	1.22	9	0.13^**^	0.33^**^	−0.42^**^	−0.39^**^	**0.74**		
6. Cynicism	2.48	1.08	5	0.11^*^	0.21^**^	−0.48^**^	−0.41^**^	0.59^**^	**0.76**	
7. Professional realization	5.44	0.82	8	−0.07	−0.09^*^	0.29^**^	0.21^**^	−0.26^**^	−0.41^**^	**0.73**

*The reliability of the variables of the study are on the diagonal*.

* *p < 0.05*.

** *p < 0.01*.

^***^p < 0.001.

A regression analysis was performed for each of the possible combinations between the dimensions of non-physical violence and satisfaction with emotional exhaustion and for cynicism. Physical violence was omitted from the analysis, as it was not the aim of study of this paper. As can be seen, Table 3 analyzes the dimension of cynicism through a regression analysis in which the first block of variables corresponds to non-physical violence and extrinsic satisfaction, and the second block corresponds to non-physical violence and intrinsic satisfaction. These variables are significantly related to cynicism, although in the case of non-physical violence, the relationship was positive and, for extrinsic and intrinsic satisfaction, it was negative. The interaction of non-physical violence and extrinsic satisfaction was significant and negative (β = −0.444, *p* < 0.01), explaining 17% of the variance in cynicism. Likewise, the interaction of non-physical violence and intrinsic satisfaction was negative, explaining 18.51% of the variance in cynicism (β = −0.264, *p* < 0.05).

**Table 3:** Regression equation predicting cynicism from non-physical violence and extrinsic and intrinsic job satisfaction.

	**β**	***R* ^2^**	**Δ*R*^2^**
**Extrinsic satisfaction model**			
Non-physical violence	0.113^*^	0.169	
Extrinsic satisfaction	−0.366^***^		
Non-physical violence X Extrinsic satisfaction	−0.444^**^	0.185	0.016
**Intrinsic satisfaction model**			
Non-physical violence	0.070	0.228	
Intrinsic satisfaction	−0.449^***^		
Non-physical violence X Intrinsic satisfaction	−0.264^*^	0.236	0.008

* *p < 0.05*.

** *p < 0.01*.

*** *p < 0.001*.

Regarding the results of this same regression analysis of emotional exhaustion (Table 4), we can see that the interaction of non-physical violence and extrinsic satisfaction explained 24.9% of the variance of emotional exhaustion (β = −0.483, *p* < 0.001) (Table 4). In addition, the interaction between non-physical violence and intrinsic satisfaction explained 24.4% of the variance of emotional exhaustion (β = −0.250, *p* < 0.05).

**Table 4:** Regression equation predicting emotional exhaustion from non-physical violence and extrinsic and intrinsic satisfaction.

	**β**	***R* ^2^**	**Δ*R*^2^**
**Extrinsic satisfaction model**			
Non-physical violence	0.272^***^	0.230	
Extrinsic satisfaction	−0.328^***^		
Non-physical violence X Extrinsic Satisfaction	−0.483^***^	0.249	0.019
**Intrinsic satisfaction model**			
Non-physical violence	0.024^***^	0.237	
Intrinsic satisfaction	−0.347^***^		
Non-physical violence X Intrinsic satisfaction	−0.250^*^	0.244	0.007

* *p < 0.05*.

^**^p < 0.01.

*** *p < 0.001*.

No interaction between extrinsic and intrinsic satisfaction and non-physical violence was observed for Professional Fulfillment.

**Figure 1:**
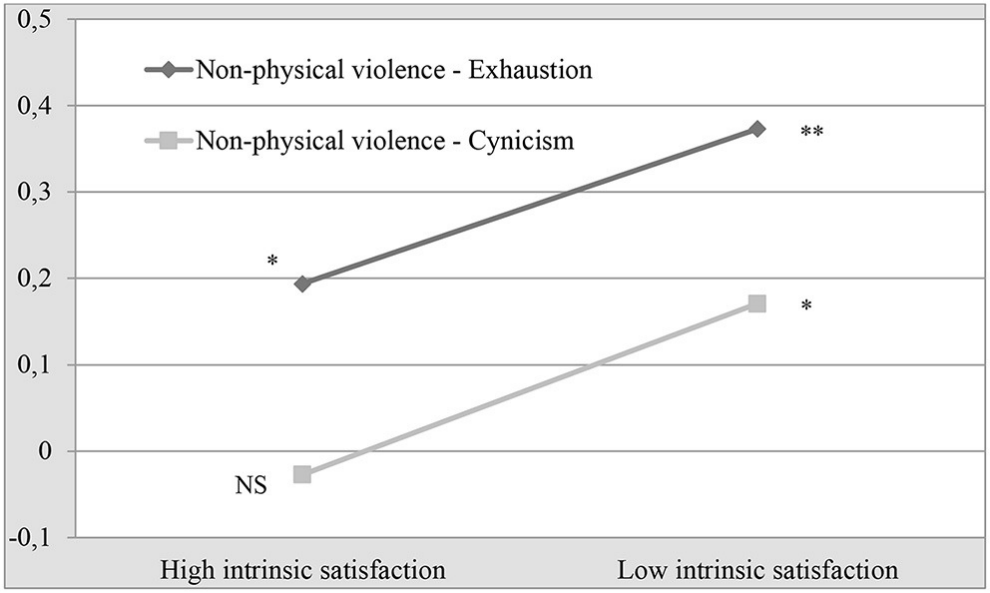
Interaction of non-physical violence and intrinsic satisfaction on the levels of exhaustion and cynicism.

**Figure 2:**
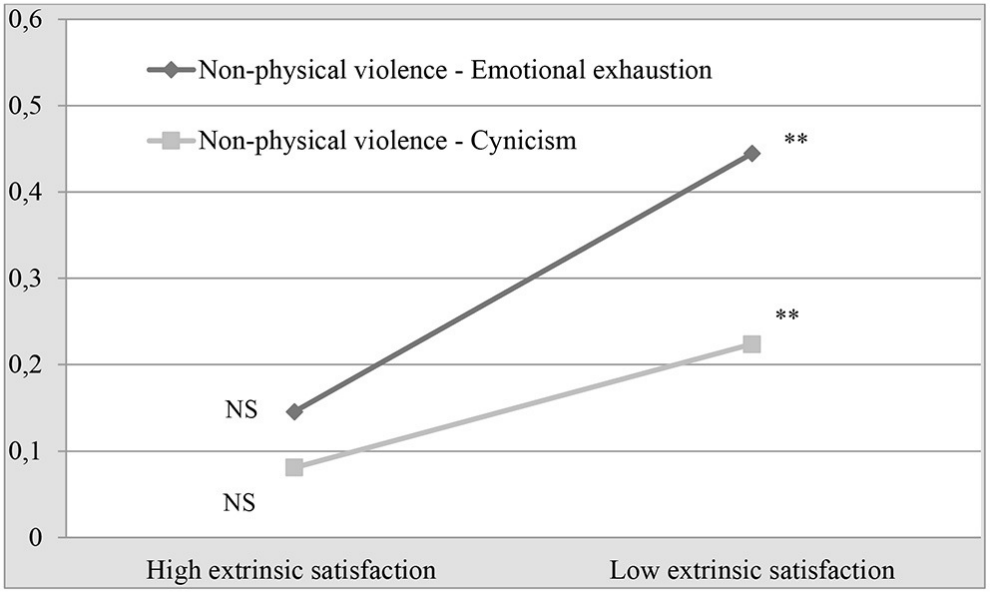
Effect of the interaction of non-physical violence and extrinsic satisfaction on the levels of emotional exhaustion and cynicism.

To elaborate on this, the interpretation of these results is represented graphically in Figures 1, 2, which show that, with low levels of satisfaction, the mean scores on emotional exhaustion and cynicism are higher for the same level of violence. Figure 1 shows that, when comparing high and low levels of exposure to non-physical violence, the difference in the mean emotional exhaustion score is higher when satisfaction is low (2.51.vs. 3.12). This is more noteworthy in cynicism, where, with high levels of intrinsic and extrinsic satisfaction, the mean cynicism score is very similar, even if the scores on non-physical violence increase (Figure 2).

## Discussion

This study provides evidence of the psychological consequences of exposure to user violence in PHC personnel. Following the objectives of this study, the results indicate that the non-physical violence perceived by professionals, especially when scores in job satisfaction are low, is relevant for the onset of the burnout syndrome in PHC staff. These results also confirm the main hypothesis of our study. The relationship between non-physical violence and burnout found is consistent with previous studies, in which non-physical violence, mainly verbal aggression, was positively associated with emotional exhaustion, cynicism and reduced professional efficacy (19, 27, 39). This association has also been observed in Primary Care (34). Hence, it is important to take these results into account, since several studies indicate a high prevalence of non-physical violence among Primary Care practitioners (14, 34).

Previous studies have explored possible variables related to conflicts and violence in Primary Care centers. In this regard, women and PHC personnel with fewer years of professional experience report greater exposure to non-physical violence (8). Furthermore, a recent qualitative study has explored the causes of conflicts in Primary Care centers (40). The aspects that seem to cause most con?icts between users and professionals from an organizational point of view are the uncertainty in waiting times, the need to adapt the telematic or telephone appointment to the different types of users, and the management of emergencies. On the other hand, regarding the professionals, users pointed out that the medical staff is perceived as distant and sometimes does not provide enough information on the health status of users (40). Among the alternatives proposed to reduce the conflict are the incorporation of specific support sta? for appointment management, the unloading of the administration service, the training of professionals in patient care and the incorporation of personnel specialized in psychosocial problems and the creation of support groups (40).

Although some authors propose burnout as a consequence of exposure to violence, others consider that workers' emotional exhaustion leads to an inappropriate management of aggressive episodes and facilitates new episodes of violence (41–43), in our opinion, the inclusion of job satisfaction in this relationship allows us to better understand this relationship and the possible differences found both between services and between professionals.

Regarding the relationship between violence and burnout, Hensel et al. (44) found that burnout is related to a high prevalence of workplace violence. In addition, fear of future violence has also been found to be positive and significantly related to cynicism and emotional exhaustion (16, 44, 45), being present in more than two out of three professionals (16). In this regard, job control has been found to be a moderating variable in the relationship between fear of future violence and emotional exhaustion, in such a way that the relationship was stronger for those with low job control (45). Nonetheless, this moderation between fear of future violence and cynicism was not found (45). Furthermore, the support of the administration functions has been found to be a modulating factor of aggression (11). These job-related variables (work control, workload, work demands and work climate) are, in our opinion, closely related to job satisfaction, and our results may be partially in agreement with those reported by other authors.

In this line, in our study, the two dimensions of job satisfaction are significantly and negatively correlated with burnout, coinciding with previous findings in which job satisfaction has been found to be strongly associated with the three components of burnout (46). We have also found a significant correlation between satisfaction and exposure to violence, in line with other studies (26). It is important to take this last relationship into consideration, as several studies indicate that the presence of burnout is frequent in health care professionals (15, 18, 47).

In our study, in order to further examine the nature of this relationship, we conducted a regression analysis, concluding that both extrinsic and intrinsic satisfaction modulate the relationship between non-physical violence, emotional exhaustion and cynicism. Nevertheless, these results cannot fully explain the onset of burnout syndrome. Specifically, participants with similar exposure to non-physical violence, but with low levels of job satisfaction, had higher levels of exhaustion and cynicism. On the other hand, it is observed that, with high levels of extrinsic and intrinsic satisfaction, professional exposed to high levels of violence show lower scores in emotional exhaustion and cynicism. Thus, we could consider job satisfaction as a possible protective factor for psychological health in workers exposed to non-physical violence. In this regard, it has been found that emotional regulation, positively correlated to job satisfaction (30), strengthened the role of gratitude as a buffer against the effect of cognitive change on emotional exhaustion and the effects on the modulation of the emotional exhaustion response and depersonalization (48). Similarly, positive work motivation was a moderator of exposure to aggression and emotional exhaustion (44).

In this way, and following the ideas proposed by Galián-Muñoz et al. (25), emotional exhaustion and cynicism seem to be not only a consequence but also a possible cause of a greater perception of exposure to violence. A possible explanatory model, following the general model of violence of Di Martino (49), there may be a circular and bidirectional relationship between exposure of violence, job satisfaction and burnout. In this sense, exposure to violence would reduce job satisfaction and would increase burnout risk. Moreover, this phenomenon has also been explained through the fear avoidance model (50), proposing that professionals who experience high levels of fear of future violence in the workplace are more likely to worry about it, leading to and resulting in a lack of trust between professionals and patients (16). Likewise, they may be more reluctant to engage and cope with the complex and difficult work they often encounter, thus associating this fear with the distancing from patients in both psychology and behavioral aspects (45), increasing the degree of cynicism (16).

## Limitations

The design of this study has some limitations that need to be taken into account, such as the establishment of causal relationships between the study variables and the use of self-informed questionnaires based on recall, which, in addition to the subjective nature of perceived violence, could bias the results. On the other hand, the sample, while representative of the majority of PHC centers in the Region of Murcia, is limited, so it would be interesting to include other communities or countries, as well as other professional groups in the future. Finally, there is a high percentage of missing socio-demographic data. Although this, theoretically, does not influence the analysis carried out, it should be taken into consideration.

## Research and Clinical Implications

The variables taken into account in this study have been addressed in different health services and professional groups of that sector (4, 12, 24, 33), finding similar relationships. This indicates that this relationship is transversal to the health system and, therefore, could serve as a basis for future intervention plans, especially in PHC. Such plans could address the problem of user violence, taking into account the psychological aspects of the professionals studied herein, along with other directly related aspects, such as communication skills, which could help professionals to feel more secure and competent, thus favoring interpersonal relationships with patients (51).

These results allow us to hypothesize that, the more satisfied and healthier the health care professional feels, the lower the probability of receiving violence and, at the same time, the professional will offer a better user experience. Hence, work focused on reducing the risk of user violence, as well as interventions focused on the prevention of work situations that favor burnout symptoms, would improve the professionals' satisfaction and, therefore, the care received by the users.

## Data Availability Statement

The raw data supporting the conclusions of this article will be made available by the authors, without undue reservation.

## Ethics Statement

The studies involving human participants were reviewed and approved by Servicio Murciano de Salud. The patients/participants provided their written informed consent to participate in this study.

## Author Contributions

DP, PL-Z, JR-H, EP-L, and IG-M: conceptualization, methodology, formal analysis, and investigation. DP and JR-H: validation. DP, PL-Z, RL-L, JR-H, EP-L, and IG-M: writing—original draft preparation. DP, PL-Z, RL-L, JR-H, EP-L, IG-M, and BM-J: writing—review and editing. DP and BM-J: funding acquisition. All authors have read and agreed to the published version of the manuscript.

## Conflict of Interest

The authors declare that the research was conducted in the absence of any commercial or financial relationships that could be construed as a potential conflict of interest.

## Publisher's Note

All claims expressed in this article are solely those of the authors and do not necessarily represent those of their affiliated organizations, or those of the publisher, the editors and the reviewers. Any product that may be evaluated in this article, or claim that may be made by its manufacturer, is not guaranteed or endorsed by the publisher.
